# Optimization of broadband metamaterial absorber using twin delayed deep deterministic policy gradient reinforcement learning technique

**DOI:** 10.1038/s41598-026-41716-8

**Published:** 2026-04-18

**Authors:** Basant E. Mahmoud, Tamer A. Ali, Salah S. A. Obayya, Mohamed Farhat O. Hameed

**Affiliations:** 1 Communications and Information Engineering program, Zewail City of Science, Technology, and Innovation, October Gardens, 6th of October City, 12578 Giza, Egypt; 2 Mathematics and Engineering Physics Department, Faculty of Engineering, Cairo University, 12613 Giza, Egypt; 3 Centre for Photonics and Smart Materials, Zewail City of Science, Technology, and Innovation, October Gardens, 6th of October City, 12578 Giza, Egypt; 4 Faculty of Engineering, University of Mansoura, 35516 Mansoura, Egypt; 5 Center for Nanotechnology, Zewail City of Science, Technology, and Innovation, October Gardens, 6th of October City, 12578 Giza, Egypt

**Keywords:** Metamaterial absorbers, Reinforcement learning, Twin Delayed Deep Deterministic Policy Gradient model, Ultrathin wideband absorbers, Engineering, Materials science, Mathematics and computing, Optics and photonics, Physics

## Abstract

This paper presents a new reinforcement learning (RL)-driven inverse design strategy that leverages the Twin Delayed Deep Deterministic Policy Gradient (TD3) algorithm for the efficient optimization of photonic structures, with a focus on metamaterial absorbers (MAs) and cross polarization converters (CPC) as demonstrative applications. Unlike conventional heuristic or surrogate-based optimization methods, the proposed RL approach autonomously learns the optimal geometric configuration through direct interaction with the simulation environment, without requiring gradient information or pre-built surrogate models. Initially, the TD3 model is used to optimize the geometric parameters of an existing MA based on an L-shaped resonator, significantly enhancing its absorption performance to be greater than 90% in the frequency range from 12.2  GHz to 22.4  GHz in only 23 iterations. Then, a novel CPC design is proposed, optimized using the same RL framework, and subsequently fabricated. The fabricated structure achieves high polarization conversion ratio (PCR) above 90% over a wide frequency range from 11.8  GHz to 24.2  GHz, covering the full Ku band and most of the K band. Furthermore, over most of the frequency range, the converter maintains strong performance under oblique incidence, with PCR levels above 80% up to an angle of 50$^\circ$. These results validate the effectiveness of the TD3-based RL framework in discovering high-performance and fabrication-ready designs, while also establishing a scalable and generalizable optimization paradigm for advanced photonic devices.

## Introduction

Photonic devices, including waveguides, photonic crystals, modulators, and metamaterials, play a crucial role in modern optical communication^1^, sensing^2^, and energy harvesting systems^3^ due to their ability to manipulate light at subwavelength scales. Among these platforms, metamaterials have emerged as a particularly powerful class of engineered structures capable of exhibiting electromagnetic properties not found in natural materials. Their ability to achieve negative permittivity and permeability–and consequently a negative refractive index^4,5^–has enabled a wide range of advanced applications. Among these developments, metamaterial absorbers have attracted significant interest due to their capability to achieve near-perfect absorption of incident electromagnetic waves within a compact footprint^6^.

The concept of the metamaterial absorber was first introduced by Landy et al. in 2008^6^, demonstrating that perfect absorption can be achieved by simultaneously matching the impedance of free space and suppressing transmission. Different structural classes of MAs have been explored to meet diverse application requirements. In the microwave regime, metallic resonant metasurfaces composed of patterned metallic elements, such as split-ring resonators connected by copper bars, have been shown to achieve broadband absorption above 90 % over targeted frequency ranges with polarization and angle robustness^7^. Design strategies such as multilayer structures, frequency selective surfaces (FSS), and arrays of multi-sized resonators have been developed to tailor wideband responses while maintaining polarization insensitivity and robust performance at oblique incidence^8^. For example, a recent Ku-band broadband metamaterial absorber was designed and experimentally validated to provide absorptivity greater than 90 % from approximately 12.5 GHz to 14.3 GHz, with a simple unit-cell layout that is easy to fabricate and suitable for different applications^9^. At terahertz frequencies, an ultra-broadband metal–dielectric–metal absorber featuring a star-shaped metallic patch, intermediate dielectric layer, and metallic ground plane has been shown to deliver high absorption peaks (up to  99 %) at multiple resonances spanning  2.42–6.11 THz, with polarization-independent performance and appreciable absorption (>$70\,\%$) up to $45^\circ$ incidence, illustrating the extension of metallic resonant designs to the THz band^10^. Another W-band (75–110 GHz) metallic absorber using a sandwich structure with varied metal patch sizes on an FR-4 substrate achieves an average absorption exceeding 94 % across the entire band, remains polarization-insensitive and effective at oblique incidence, and demonstrates the potential of multilayer patch designs for millimeter-wave broadband absorption^11^. However, because of the high ohmic losses, low melting points, and high thermal conductivities of metals, the applicability of metal–dielectric metasurface-based perfect absorbers is limited in some cases.

In parallel, all-dielectric metamaterial absorbers have emerged as low-loss alternatives that leverage high-index dielectric resonances and careful geometric design to achieve broadband performance. In the terahertz regime, experimental demonstrations have shown that hybrid all-dielectric metasurface absorbers based on overlapping electric and magnetic dipole resonances can reach absorption levels as high as  97.5 %, offering a promising route for broadband THz absorber design^12^. Meanwhile, novel all-dielectric structures composed of fully doped silicon elements such as square rings and double H-shaped resonators have been proposed to achieve ultra-wideband THz absorption (>90 %) over the range from approximately 0.98 THz to 6.37 THz with polarization independence and wide-angle stability, enabled by synergistic multi-modal coupling and optimized impedance matching^13^. In the microwave band, transparent bi-directional water-based all-dielectric absorbers achieve >90 % absorptance across 5.7–41.6 GHz with excellent polarization insensitivity and angular robustness, highlighting the versatility of dielectric resonances for achieving ultra-wideband performance across frequency scales^14^. These varied all-dielectric MA designs illustrate the breadth of approaches being pursued to tailor absorption characteristics across frequency bands and applications.

Beyond absorption, metamaterial metasurfaces have also been extensively engineered to realize polarization control functionalities, particularly through metamaterial cross-polarizers , which convert an incident linearly polarized wave into its orthogonal polarization in reflection mode^15^. In reflective cross-polarizers, the typical metal–dielectric–metal configuration suppresses transmission using a metallic ground plane, while anisotropic or chiral resonant elements introduce a controlled phase and amplitude imbalance between orthogonal field components, enabling strong polarization rotation upon reflection^16^. The polarization conversion performance is commonly quantified using the co-polarized and cross-polarized reflection coefficients (e.g., $S_{xx}$ and $S_{yx}$), as well as the polarization conversion ratio (PCR), which approaches unity when cross-polarized reflection dominates the response^17^. Such reflective cross-polarization converters have attracted increasing attention due to their key roles in polarization multiplexing and isolation for wireless and satellite communication systems, radar stealth and radar cross-section (RCS) reduction, and wavefront-engineered sensing and imaging platforms^18–20^. However, the fabrication of these devices often involves complex nanostructures and precise material deposition, leading to high costs and stringent manufacturing tolerances^21^. Consequently, efficient optimization algorithms are essential to design photonic components that maximize performance while minimizing fabrication complexity and resource consumption.

Traditional optimization methods, such as gradient-based techniques and topology optimization, have been effectively applied to the design of photonic devices^22,23^. For example, Hammond et al.^23^ enabled the design of manufacturable integrated photonic circuits using topology optimization with fabrication constraints. In addition, metaheuristic algorithms, such as genetic algorithms (GA) and particle swarm optimization (PSO), have gained popularity for their global search capabilities in complex design spaces. In this context, Hameed et al.^24^ employed PSO and Central Force Optimization (CFO) algorithms in an innovative way to optimize the dispersion characteristics of photonic crystal fibers (PCF) and obtained zero-dispersion over a wide band of wavelengths. Furthermore, Goncalves et al.^25^ used a multisymmetric level optimization (MSL) procedure that uses GA to optimize three-dimensional photonic coupler devices. This approach effectively reduced computational requirements by dividing the device optimization into symmetric regions. Furthermore, Hameed et al.^26^ introduced a modified derivative-free surrogate-based trust region optimization algorithm tailored to optimize PCF dispersion properties to achieve a highly negative flat dispersion. In addition to photonic device optimization, extensive efforts have also been devoted to applying traditional and metaheuristic optimization algorithms to the design of MAs. For example, Pelluri and Appasani^27^ employed a GA to optimize the unit-cell geometry of an X-band metamaterial absorber, achieving over 90 % absorption across much of the X-band and a peak of nearly 99.95 % at 10.52 GHz. Hammad et al.^28^ used a multilevel coordinate search (MCS) with a kriging surrogate model coupled to finite-element simulations to optimize an ultrathin terahertz metamaterial absorber with a discontinuous gold resonator on a lossy dielectric, achieving over 90 % absorption from 0.97 to 3.76 THz with high relative absorption bandwidth, polarization insensitivity, and strong performance over large incident angles. Similarly, ultra-broadband MA designs spanning from ultraviolet to mid-infrared wavelengths have been reported by Fuyin Luo^29^, where careful engineering of multilayer metal–dielectric structures and resonator geometries yields continuous high absorption over extended spectral ranges using a simulated annealing algorithm, illustrating the potential of broadband absorber optimization strategies. Furthermore, Peng et al.^30^ used a material–structure integrated design (MSID) approach, combining a genetic algorithm with full-wave simulation to co-optimize dielectric materials and unit-cell geometry for ultra-broadband all-dielectric absorption from 5.3 to 18 GHz, demonstrating how systematic optimization of both material and structure can excite multiple resonances for broadband performance . In another example, Shi et al.^31^ applied topology optimization to a water-based all-dielectric metamaterial absorber, using the heights of discretized water columns as design variables to iteratively adjust impedance matching and resonant features, yielding a very high average absorption rate across the 9–22 GHz band. Despite these advances, traditional and metaheuristic methods often suffer from slow convergence, high computational demand, and sensitivity to initial conditions, which limits their scalability to high-dimensional photonic device design problems^32^. Therefore, there is a need for novel optimization techniques that can overcome these challenges and enable an efficient and accurate design of photonic devices.

In recent years, artificial intelligence (AI) has emerged as a transformative tool for photonic device design^33^. AI encompasses computational techniques that enable machines to perform tasks traditionally that require human intelligence. However, machine learning (ML) is a domain within AI focused on algorithms that learn from data to make predictions or decisions without explicit programming^34^. Deep learning (DL), a further specialization of ML, uses multi-layered neural networks to automatically extract hierarchical features from large and complex datasets. Therefore, a breakthrough in image and speech recognition is achieved using the DL technique^35^. Reinforcement learning (RL) is a subset of AI in which an agent learns to make sequential decisions by interacting with an environment and maximizing cumulative rewards. RL differs from supervised learning that relies on labeled data and unsupervised learning which identifies patterns without explicit feedback^36^.Unlike DL, RL learns optimal policies through trial and error interactions with an environment, receiving feedback in the form of rewards or penalties.

Recent years have witnessed the application of AI, ML, and RL techniques to optimize photonic devices with promising results. For example, Gaussian Process Regression (GPR) models have been used to optimize photonic crystal cavities, effectively accounting for fabrication uncertainties and leading to a more robust device performance^37^. Deep reinforcement learning (DRL) has been applied to the inverse design of photonic crystals for nanoscale laser cavities, enabling automated discovery of high-quality designs that outperform traditional methods by 2 orders and by 10 times improvement in performance compared to humans^38^. In multi-wavelength optical systems, DRL calibration methods have been introduced to address frequency-selective responses caused by fabrication and environmental factors, resulting in improved signal processing capabilities^39^. Furthermore, interpretability techniques like Local Interpretable Model-Agnostic Explanations (LIME) have been integrated into ML-based inverse design processes, providing insights into design-performance relationships and guiding better initial conditions for optimization^40^. Collectively, these studies underscore AI’s potential to overcome traditional optimization limitations in photonics. A notable recent study by Gamal et al.^41^ employed the Advantage Actor-Critic (A2C) RL model to optimize photonic structures through interaction with simulation environments. While this work successfully demonstrated RL’s applicability to photonic inverse design, it was limited by the use of a discrete action space, which is less suitable for inherently continuous photonic design parameters, potentially leading to coarse approximations and suboptimal solutions. Continuous action space RL models, such as those based on the Twin Delayed Deep Deterministic Policy Gradient (TD3) algorithm, provide smoother policy updates and finer control over continuous variables, making them more appropriate for photonic device optimization.

In this study, we present a novel RL-based framework for the design and optimization of a broadband Metamaterial absorber (MA) using the TD3 algorithm. It is worth noting that the reinforcement learning has been applied in various metamaterial design contexts^41,42^. However, the previously reported structures have been optimized using other models as double deep Q-learning network (DDQN)^42^ and Actor-Critic^41^. In contrast, the present work emphasizes the use of the TD3 algorithm for optimization within a continuous action space, which offers distinct advantages over discrete-action RL approaches such as A2C. The TD3 has proven to be highly effective for continuous control tasks^43^. Beyond metamaterial design, TD3 has been effectively employed in a variety of continuous optimization contexts, such as for robust controller tuning in power electronics to regulate DC microgrid converters^44^, optimizing three-dimensional UAV trajectories and communication parameters in downlink wireless systems^45^, and enhancing collision-free motion planning for robotic navigation in complex environments^46^, demonstrating its broad applicability to continuous control problems across different engineering domains. It builds upon the Deep Deterministic Policy Gradient (DDPG) framework by addressing overestimation bias through twin Q-networks and improving training stability via delayed policy updates and target smoothing^43^. This robustness makes TD3 an ideal candidate for high-dimensional, continuous parameter optimization problems such as those encountered in MA design. The proposed approach involves defining the absorber structure within commercial simulation technology (CST) Microwave Studio software, parameterizing key geometric variables, and allowing the TD3 agent to interact with the simulation environment. The agent receives absorption spectra as a feedback and iteratively adjusts the design parameters to maximize the absorption over a target frequency range. It is used to fine-tune the geometry of an ultra-thin, single-layer MA. This approach enabled the design of a structure with high absorptivity (exceeding 90%) across a broad frequency range from 12.2 to 22.4 GHz, effectively spanning much of the Ku and K bands. To assess the effectiveness of the algorithm, the optimized results are compared with a previously published design using the same structure^47^. The achieved improvement confirmed the potential of the proposed method for enhancing the MA performance. Then, the algorithm is subsequently applied to develop a novel, simplified single-layer CPC devoid of lumped elements. This innovative design achieved high PCR (above 90%) over a broadband frequency span from 11.8 to 24.2 GHz, covering the entire Ku band (12–18 GHz) and most of the K band (18–27 GHz). Remarkably, the converter maintained oblique incidence stability with PCR above 80% throughout most of the frequency band for incident angles up to $50^\circ$, indicating robust angular stability. Additionally, the proposed design has been fabricated, and the prototype has undergone experimental characterization, with measured results aligning closely with simulations, thereby corroborating the design’s reliability. Finally, a comparative analysis with existing designs has been performed to underscore the performance of the introduced structure in terms of bandwidth, PCR ration, and compactness. This work offers a generalizable framework that can be extended to optimize other photonic or microwave components.

The remainder of this paper is structured as follows: Section 2 describes the TD3 algorithm and its customization for the optimization task. Section 3 discusses the results and compares them with state-of-the-art designs. Section 4 concludes the study with observations and results.

## Mathematical formulation of TD3 model

Optimizing metamaterial absorbers, designed to achieve near-unity absorption over specific frequency ranges, presents a complex challenge due to the high-dimensional and continuous nature of the design space. Traditional optimization methods often struggle with the non-convexity and multimodality inherent in such problems. To address these challenges, we employ the TD3 algorithm, a state-of-the-art RL method tailored for continuous control tasks.

**Fig. 1:**
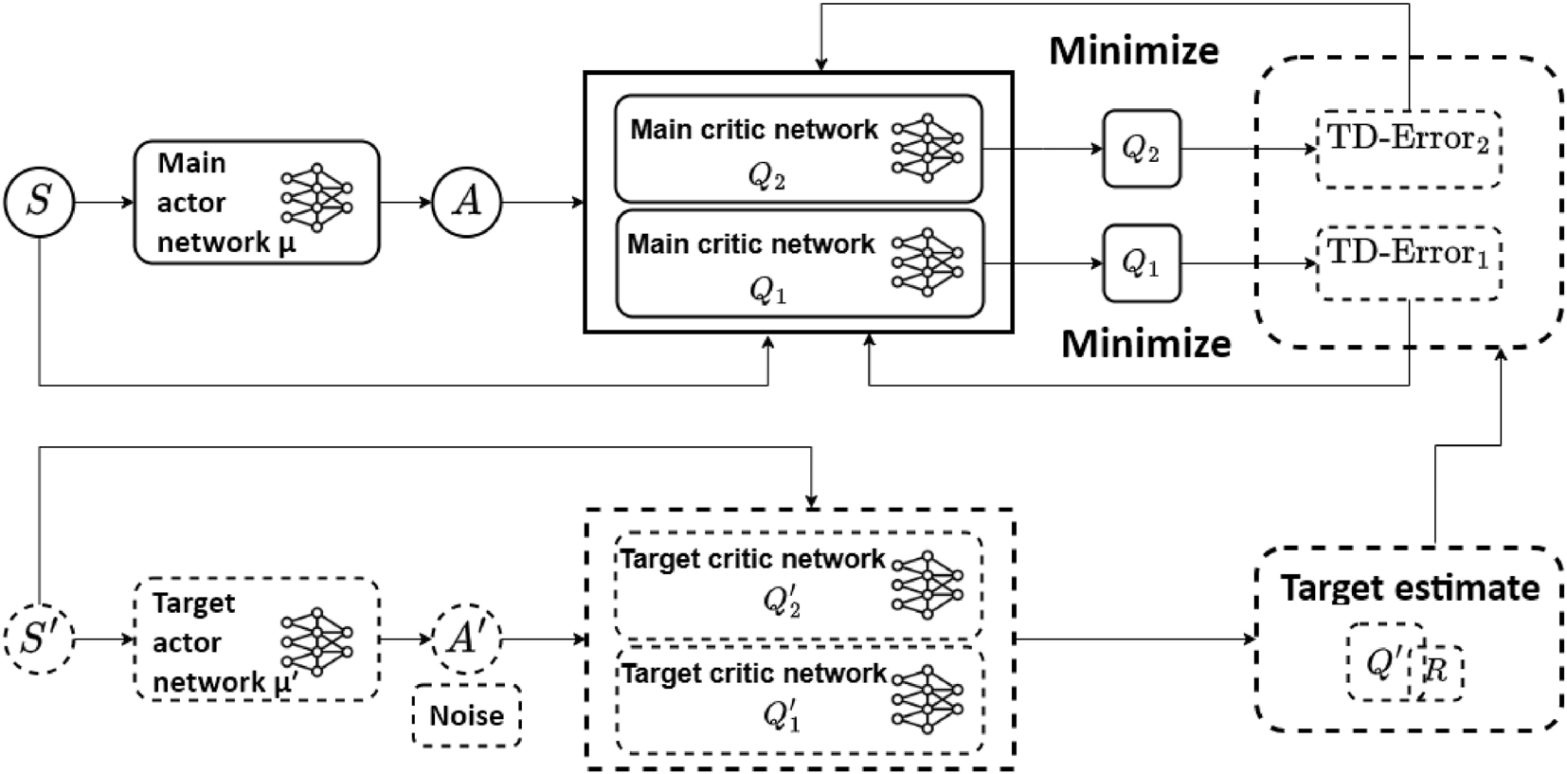
Schematic diagram of TD3 structure.

TD3 is an off-policy, actor-critic algorithm that extends the DDPG framework by incorporating several key enhancements to improve learning stability and performance^43^. These enhancements include the use of twin critic networks to mitigate overestimation bias, delayed policy updates to stabilize training, and target policy smoothing to prevent the exploitation of sharp value function peaks. Figure  1 shows the flow of the TD3 algorithm.

For the present optimization problem, the interaction between the TD3-RL agent and the environment can be described in terms of states, actions, and rewards. The state denotes the current configuration of the MA, characterized by its geometric parameters such as the thickness, spacing, and length of the resonator elements. An action represents a modification of these parameters within the continuous design space, which is subsequently applied to the environment, modeled by the full-wave electromagnetic simulator (CST). For example, when the state consists of a set of geometric parameters, the corresponding action represents updated values of these parameters within their allowable ranges. The simulator evaluates the updated design by computing its absorption spectrum, from which the reward is determined. This reward quantifies the absorption performance of the structure, with higher values assigned to configurations that achieve broadband and high absorption within the target frequency range. The simulator then provides the agent with the corresponding new state, thereby completing one interaction cycle.

In the TD3 framework, the agent interacts with an environment characterized by a state space $\mathcal {S}$, an action space $\mathcal {A}$, with respect to a deterministic policy $\pi _{\phi }: \mathcal {S} \rightarrow \mathcal {A}$, parameterized by $\phi$, receiving a reward $r$ and new environment state $s'$. In contrast to discrete action reinforcement learning – where the action space $\mathcal {A}$ is a finite set of predefined choices – in this context, the action space is defined as a continuous space:

$$\mathcal {A} = \mathbb {R}^{n}$$

The objective is to maximize the expected cumulative reward^43^:

1 $$\begin{aligned} \mathbb {E}_{s_t \sim \mathcal {D}, \, a \sim \pi _\phi } \left[ \sum _{t=0}^T \gamma ^t \, r(s_t, a_t) \right] , \end{aligned}$$

where $\gamma \in (0,1]$ is the discount factor that determines the relative importance of short-term versus long-term rewards, and $\mathcal {D}$ denotes the replay buffer containing past experiences.

To address overestimation bias, TD3 employs two critic networks $Q_{\theta _1}$ and $Q_{\theta _2}$, and uses the minimum of their estimates to compute the target Q-value. This conservative estimate leads to a more stable and reliable Clipped Double Q-learning target:

2 $$\begin{aligned} y = r + \gamma \cdot \min _{i=1,2} Q_{\theta '_i}(s', \pi _{\phi '}(s') + \epsilon ), \end{aligned}$$

where $\theta '_i$ and $\phi '$ are the parameters of the target networks, and to prevent the policy from exploiting sharp peaks in the value function, TD3 adds noise to the target action during the critic update. This technique, known as target policy smoothing, involves adding clipped noise $\epsilon \sim \text {clip}(\mathcal {N}(0, \sigma ), -c, c)$ to the action selected by the target policy. This regularization encourages the policy to be robust to slight perturbations, leading to smoother value estimates and improved performance.

The critic networks are updated by minimizing the loss:

3 $$\begin{aligned} L(\theta _i) = \mathbb {E}_{(s,a,r,s') \sim \mathcal {D}} \left[ \left( Q_{\theta _i}(s,a) - y \right) ^2 \right] , \end{aligned}$$

for $i = 1, 2$. In TD3, the policy (actor network) and target networks are updated less frequently than the critic networks. Specifically, for every $d$ updates of the critic networks, the actor and target networks are updated once. This delay allows the critic networks to converge to more accurate value estimates before the policy is updated, thereby reducing the variance and instability in learning. The actor network is updated every $d$ steps, by maximizing the expected Q-value. In continuous–action problems, the parameterized policy $\pi _{\phi }$ is updated by evaluating the gradient of the expected return, expressed as $\nabla _{\phi } J(\phi )$:

4 $$\begin{aligned} \nabla _{\phi } J(\phi ) = \mathbb {E}_{s \sim \mathcal {D}} \left[ \nabla _a Q_{\theta _1}(s,a) \big |_{a=\pi _{\phi }(s)} \nabla _{\phi } \pi _{\phi }(s) \right] . \end{aligned}$$

The target networks are updated using a soft update mechanism:

5 $$\begin{aligned} \theta '_j \leftarrow \tau \theta _j + (1 - \tau ) \theta '_j, \quad \phi ' \leftarrow \tau \phi + (1 - \tau ) \phi ', \end{aligned}$$

where $\tau$ is a small constant ensuring gradual updates.

In summary, Table 1 presents the pseudocode for the TD3 algorithm’s training process.

**Table 1:**
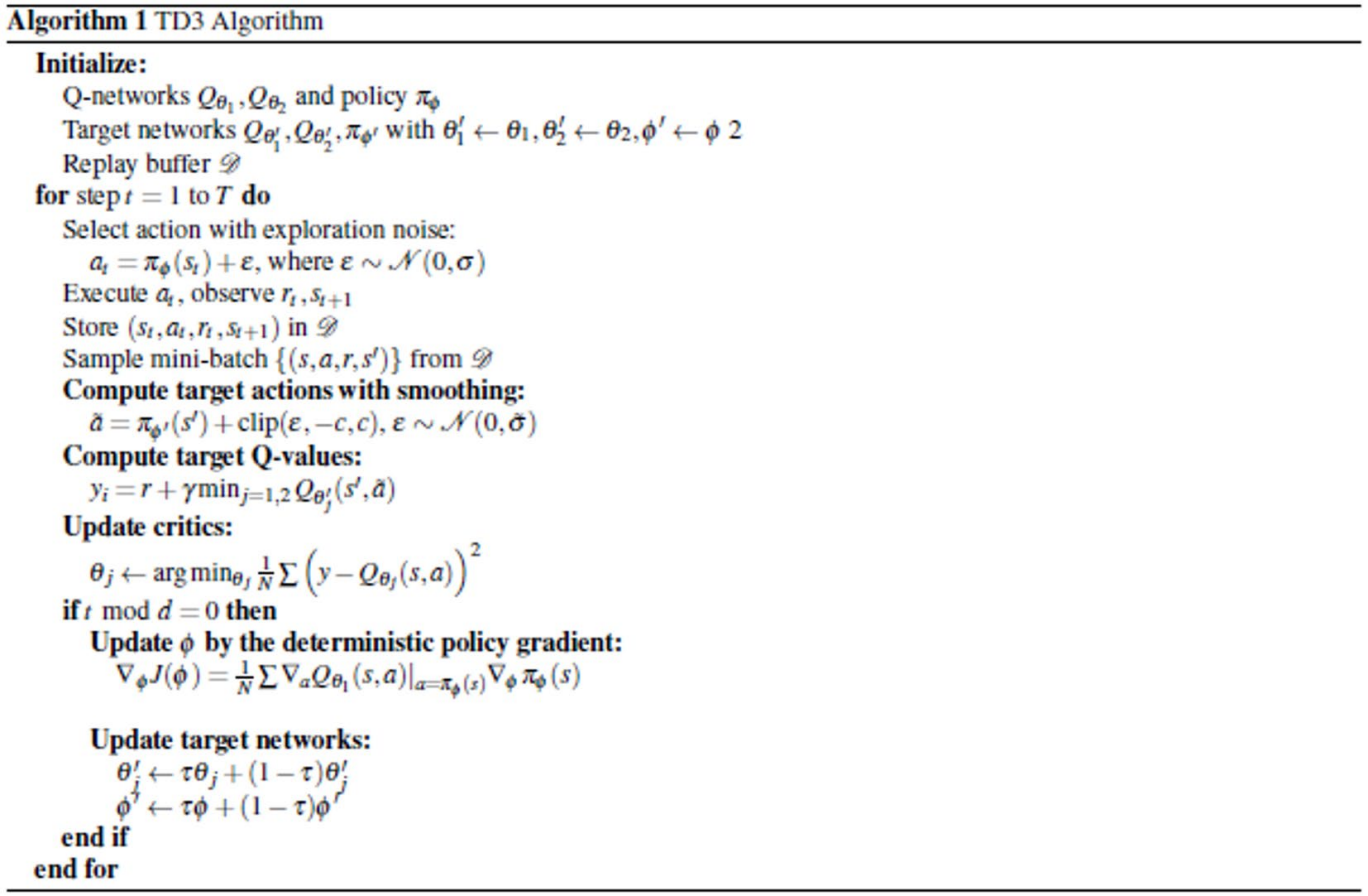
The TD3 pseudocode training process Algorithm.

In optimizing the metamaterial absorber, the reward function is crafted to align the agent’s objective with the desired absorption characteristics. Specifically, the goal is to maximize the proportion of the frequency spectrum where the absorber exhibits high performance (absorption above 90%). Although reward functions can be formulated in various ways and alternative definitions may offer additional insights, in this work the reward function defined in formula 6 is adopted due to its simplicity and ease of interpretation. This function uses the L1 norm of the deviation between the normalized absorption and the target value 0.99, which linearly penalizes deviations and provides a straightforward measure of how closely the agent meets the desired absorption characteristics.

6 $$\begin{aligned} R = -1000 \times |y - 0.99|, \end{aligned}$$

where $y$ represents the fraction of the frequency spectrum with absorption exceeding 90%. The target value of 0.99, rather than 1.0, was selected to reflect a practically achievable design goal, acknowledging that perfect absorption across the entire frequency band is physically and experimentally unrealistic. This formulation ensures that the reward is maximized when $y$ approaches 0.99, reflecting near-perfect absorption across the target spectrum. In addition, in this reward function, the agent relies on the relative differences between the reward value and the target rather than on the absolute reward magnitude, so the use of negative rewards does not hinder the optimization process.

The use of a large negative scaling factor (-1000) serves to amplify the penalty for deviations from the target absorption performance. This steep penalty gradient enhances the sensitivity of the learning process, compelling the agent to prioritize configurations that closely meet the design objective. Such an approach accelerates convergence and improves the robustness of the optimization process. Moreover, an alternative scaling factor (-10) was also tested for comparison, and the outcome of this trial is presented in the Results section. The convergence of the reward function toward higher values becomes evident after a sufficiently large number of iterations, reflecting the agent’s stable optimization behavior.

The performance of the TD3 algorithm is influenced by several hyperparameters, including learning rates, discount factor, policy update delay, and soft update coefficient. A comprehensive table detailing the specific values used in this study is provided in Table 2.

**Table 2:** TD3 hyperparameters for metamaterial absorber optimization.

Hyperparameter	Value
Critic Learning Rate	*0.001*
Actor Learning Rate	*0.0001*
Discount Factor ($\gamma$)	*0.99*
Policy Update Delay ($d$)	*2*
Soft update coefficient ($\tau$)	*0.005*

These parameters were selected based on commonly recommended values in the literature^43^, except the actor learning rate, which was reduced to 0.0001 in this study to enhance stability. The critic learning rate follows the standard value of 0.001 to enable rapid value updates and effective feedback to the actor, while the actor learning rate is reduced to 0.0001 to ensure conservative and stable policy updates^48,49^. The discount factor $\gamma$= 0.99 favors long-term rewards, encouraging the agent to consider sequences of actions that lead to high-performance absorption across the frequency spectrum. Lastly, the soft update coefficient $\tau$=0.005 ensures that the target networks change gradually, providing consistent learning targets and preventing oscillations. Together, these parameters form a well-tuned configuration that accelerates the agent’s ability to reach an optimal solution within a limited number of training iterations, which is especially important given the high computational cost of evaluating each candidate design in the photonic simulation environment.

## Results and discussion

**Fig. 2:**
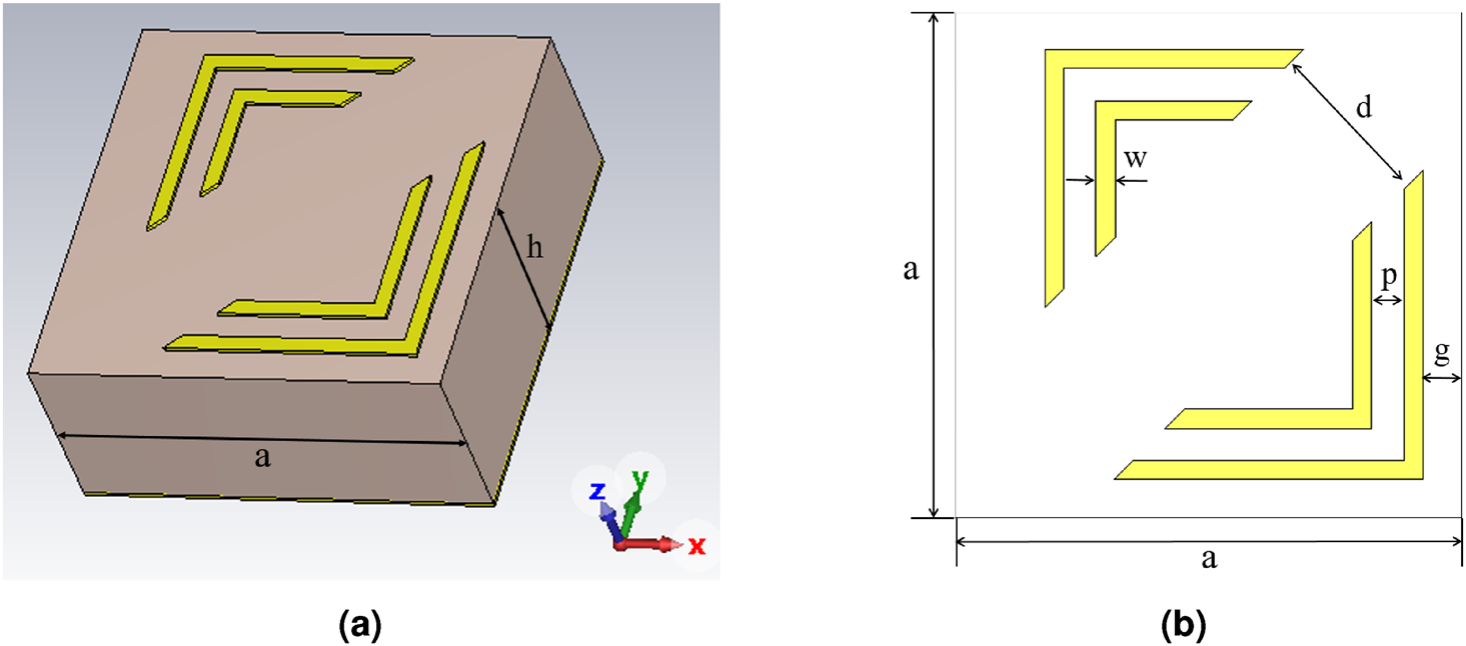
L-shaped MA design representations^47^; (**a**) Three-dimensional perspective view and (**b**) Two-dimensional cross section.

To validate the effectiveness of the proposed optimization algorithm, the structure previously fabricated and reported in Ref.  ^47^ is selected for initial evaluation. The reported MA is composed of L-shaped copper resonators and a continuous copper ground plane that ensures zero transmission, isolated by a dielectric spacer. The copper layers have a thickness of $0.035~\textrm{mm}$ with a constant conductivity $\sigma = 5.96 \times 10^{7}~\mathrm {S/m}$. The dielectric spacer is made of low-cost FR-4, characterized by a relative dielectric constant $\varepsilon _r = 4.3$, loss tangent $\tan \delta = 0.025$, and a thickness $h=1.6~\textrm{mm}$. The unit cell of the referenced design is arranged periodically with a lattice constant of $a = 3.7~\textrm{mm}$. To broaden the absorption bandwidth, a structural cut of length $d$ is introduced in the resonator layer, which, along with the L-shaped resonators, facilitates charge redistribution and generates multiple electric and magnetic dipoles. The geometry and 3D configuration of the structure are illustrated in Fig. 2.

All simulations are conducted using CST Microwave Studio with the frequency domain solver. Open boundary conditions are applied along the propagation direction (z-axis), while periodic boundary conditions are enforced in the x- and y-directions around the unit cell. A tetrahedral mesh with a precision of $10^{-4}$ is used, comprising a total of 184,690 elements.

The absorption coefficient $A$ of the metamaterial absorber at a given frequency $f$ can be calculated using reflection ($S_{11}$) and transmission ($S_{21}$) coefficients by:

7 $$\begin{aligned} A = 1 - S_{11}^2 - S_{21}^2 \end{aligned}$$

Since the bottom metal layer is 0.035 mm thick –significantly greater than the skin depth– it effectively blocks transmission, making $S_{21} \approx 0$. Thus, the absorption simplifies to:

8 $$\begin{aligned} A = 1 - S_{11}^2 \end{aligned}$$

The optimization aims to realize a broadband MA. To achieve this aim, the reward function used in equation 6 is applied. By increasing the reward of the TD3-RL model, the total absorptivity of the MA across the spectrum from 10 to 25 GHz increases. The variables d, g, w, and p are selected as design parameters for the optimization. The parameters are optimized within the following ranges: d $\in [0.1, 1.5]$, p $\in [0.1, 0.31]$, g $\in [0.1, 0.31]$, and w $\in [0.1, 0.31]$. These ranges are chosen to facilitate fabrication and maintain the desired shape of the device. Additionally, 1000 frequency points are sampled across the studied range with an interval of 0.015 GHz. The TD3-RL model was running for 45 iterations, which was sufficient for the proof-of-concept stage. Given the robustness of TD3 to initial conditions, the optimization parameters were initialized randomly to ensure diverse exploration during training. The model achieved the best results after only 23 iterations. A total of 2 hours was required to complete all iterations on a Dell Precision 7820 Tower workstation with an Intel^®^ Xeon^®^ Gold 6211 CPU @ 3.60 GHz and 384 GB RAM. It should be noted that the training time is dominated by the electromagnetic simulations at each iteration rather than by the computational cost of the RL algorithm itself.

**Fig. 3:**
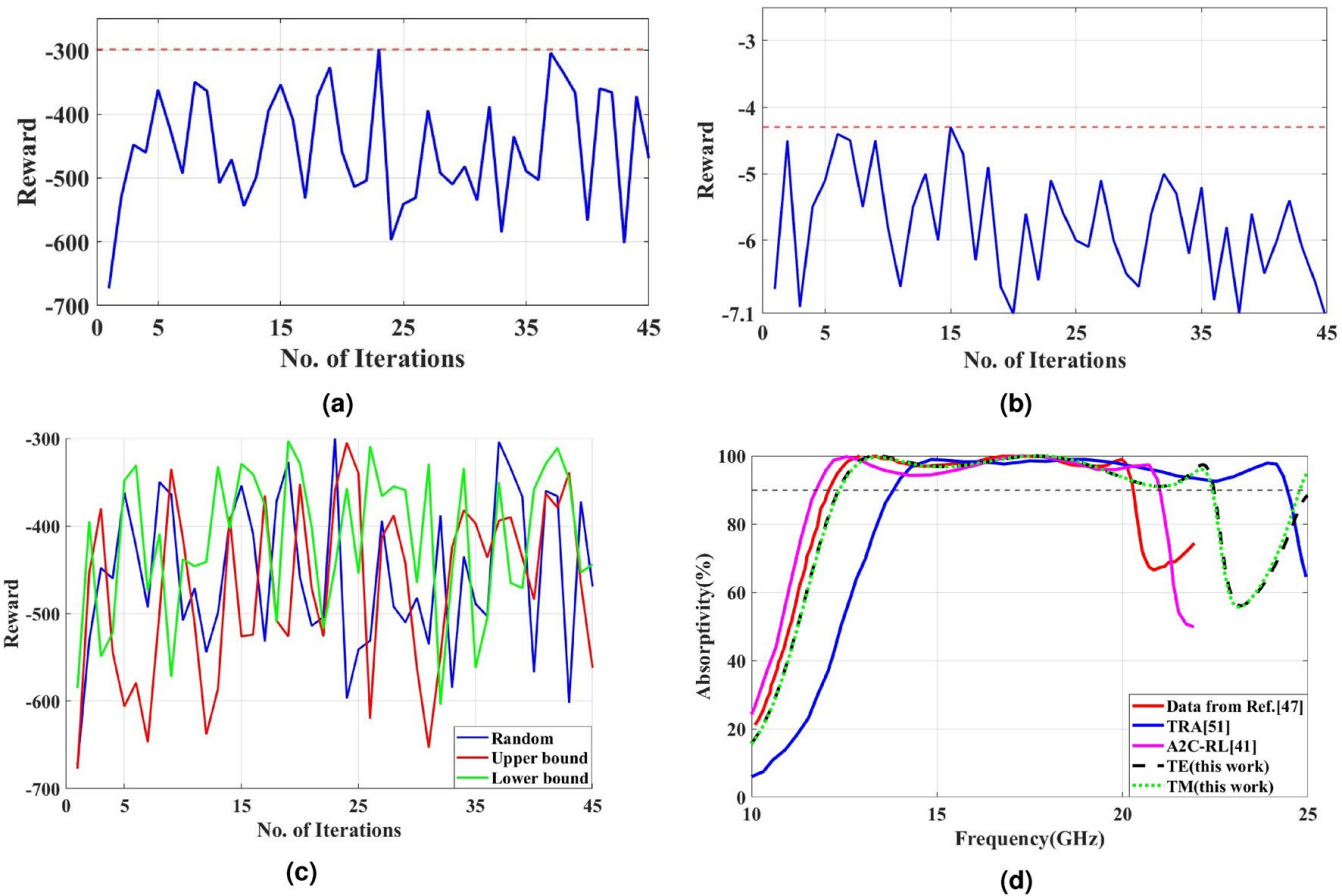
Optimization process evaluation: Training curves for reward formula versus the number of iterations with (**a**) penalty factor –1000 (**b**) penalty factor –10, (**c**) Training curves for three runs with different initializations. (**d**) Performance comparison between the optimized MA using TD3-RL model and that reported with parametric sweep in Ref. ^47^, TRA ^51^, and A2C-RL ^41^.

The model training diagram is shown in Fig. 3a which demonstrates the immediate reward value obtained at each iteration for the corresponding generated design and shows that the model achieved the maximum reward value at the 23$^{\text {rd}}$ iteration. It is worth noting that the high-performance design obtained within 23 iterations is not a random outcome but reflects the ability of reinforcement learning algorithms to efficiently identify promising solutions even during the early stages of training. This observation is consistent with previous work, where an A2C-RL model surpassed the particle swarm optimization (PSO) method in the optimization of the grating coupler in only 14 iterations ^50^. To further examine the effect of the reward formulation, an additional trial was conducted using a smaller penalty factor (-10 instead of -1000). The corresponding training curves are shown in Fig. 3b, where the y-axis scales differ due to the change in penalty magnitude. In this case, the model was unable to improve its performance with iterations and achieve a high reward value, confirming that the larger penalty scaling used in this work is necessary to guide the learning process effectively. In this study, the effectiveness of the designs is compared using the mean absorption of the MA across the frequency range through the following equation.

To further assess the stability of the optimization process, two additional runs are performed using different initializations: one initialized at the lower bounds of all geometric parameters and another at their upper bounds. As shown in Fig. 3c, all runs converged toward nearly the same reward value within a similar number of of iterations at the 19$^{\text {th}}$ and 24$^{\text {th}}$ iteration of the lower and upper bounds, respectively. This consistency across distinct starting points demonstrates the robustness of the TD3-based optimization framework to variations in the initial design state.

9 $$\begin{aligned} f(x) = \frac{\displaystyle \sum _{f=10\,\textrm{GHz}}^{f=25\,\textrm{GHz}} A(f)}{\text {number of frequencies}} \end{aligned}$$

The design parameters obtained in the 23$^{\text {rd}}$ iteration are applied and its performance is comprehensively compared against three relevant works. In this context, the optimized design using the TD3 is compared to that studied by parametric sweep in Ref. ^47^ and that optimized using A2C reinforcement learning technique (A2C-RL)  ^41^. Further, Etman et al. ^51^ have used the Trust Region Algorithm (TRA) based on Co-kriging model for the optimization of the same absorber. It is worth noting that these prior studies were applied to the exact same L-shaped absorber geometry considered in this work, ensuring a consistent and fair basis for comparison. The summarized results of these comparisons are provided in Table 3. Furthermore, Fig.  3d shows the absorption response of the optimized MA for both TE and TM polarizations compared to the results reported in the other studies. It is evident that the proposed MA demonstrates absorptivity exceeding 90% over a broader frequency range–from 12.2 to 22.4 GHz–effectively covering the majority of the $Ku$ and $K$ bands. Therefore, the TD3-RL model successfully identified a design with higher absorptivity and a wider bandwidth while maintaining the same structural configuration, thickness, and periodicity when compared to the A2C-RL and TRA models. Consequently, the TD3-RL provides a reliable and effective alternative for the inverse design of metamaterial absorbers, particularly in problems involving continuous design spaces.

**Table 3:** Design parameters and bandwidth comparison across different methods.

Parameters (mm)	Other Methods			TD3-RL Model	
	Ref. ^47^	TRA ^51^	A2C-RL ^41^	Initial	Proposed
d	0.5	1.51	0.7	1.24	**0.957**
g	0.3	0.12	0.2	0.28	**0.165**
w	0.2	0.11	0.2	0.138	**0.22**
p	0.2	0.24	0.4	0.237	**0.18**
Mean Absorptivity (%)	77.3%	81.8%	82.6%	67.7%	**85**%
Bandwidth (GHz)	12–20.2	13.8–24.5	11.7–21.2	16.4–20.7	**12.2–22.4**

**Fig. 4:**
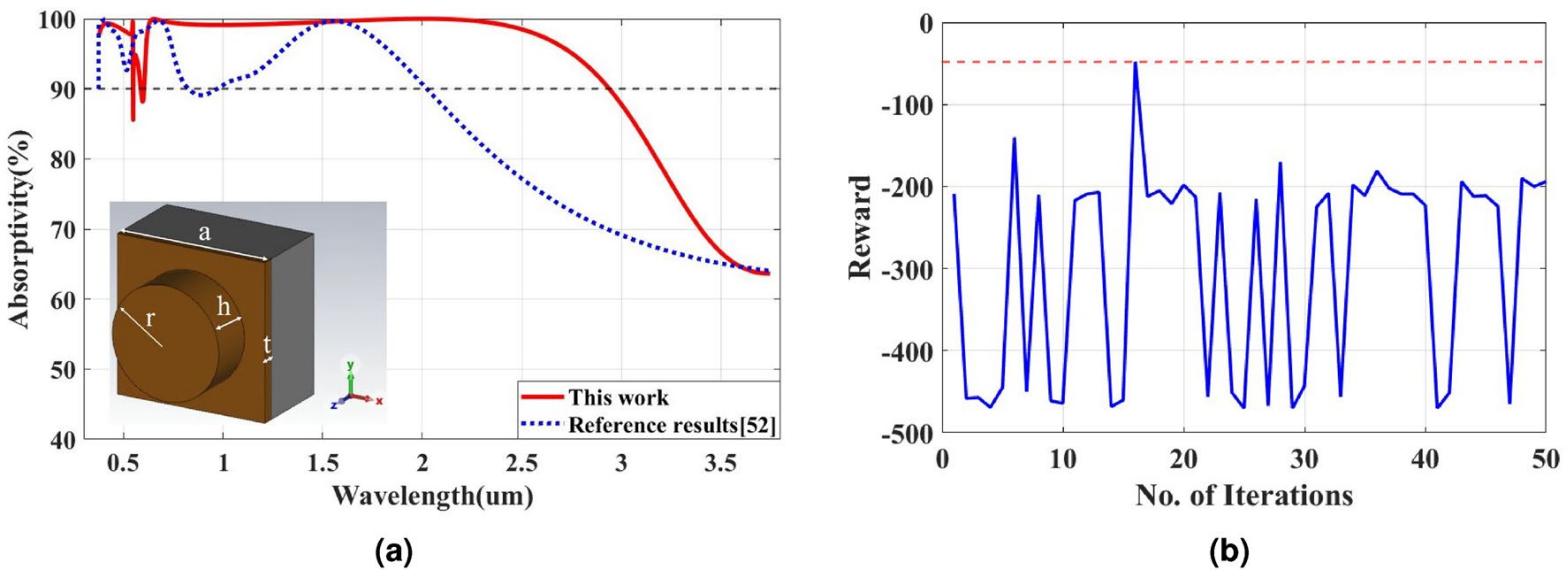
Optimization results of dielectric metasurface absorber; (**a**) absorption curves for the optimized and reference designs, where the inset figure shows 3D structural overview of the dielectric metasurface absorber, and (**b**) the training diagram of the TD3-RL model with the number of iterations.

To confirm the versatility of the proposed TD3-based optimization framework and evaluate its applicability to other classes of metamaterial absorbers, a second case study was conducted on an all-dielectric metasurface which was introduced by Cai et al. ^52^. Fig. 4a presents the schematic of the structure, consisting of a periodic array of Gallium Arsenide (GaAs) cylindrical resonators placed on a thin GaAs spacer layer and backed by a thick tungsten (W) reflector. GaAs serves as a high-index dielectric resonator capable of supporting multiple optical modes across the visible and near-infrared ranges, while the 200 nm W layer, substantially exceeding the skin depth, ensures negligible transmission. Periodic boundary conditions along the *x*- and *y*-directions and an open boundary along *z* were applied to emulate an infinite array, and the absorbance was computed using equation 8 in a frequency range from 80 to 800 THz.

The same TD3 model and reward function defined in equation 6 were applied to this dielectric metasurface, with all parameters initialized randomly to promote broad exploration of the design space. In this example, four geometric variables were selected for optimization: the cylinder radius *r*, cylinder height *h*, GaAs spacer thickness *t*, and lattice constant *a*. These parameters were optimized within the following ranges:$r \in [135, 155]~\text {nm}$, $h \in [100, 140]~\text {nm}$, $t \in [20, 40]~\text {nm}$, and $a \in [380, 420]~\text {nm}$. Using these bounds, the TD3 agent was executed for 50 iterations. Despite the material and geometric differences compared to the metallic absorber, the model rapidly adapted, achieving its highest reward at the 15$^{\text {th}}$ iteration, as illustrated in Fig. 4b. After applying the geometric parameters obtained at the 15$^{\text {th}}$ iteration, the absorption spectrum of the optimized metasurface was evaluated and compared with that of the reference design. As shown in Fig. 4a, the optimized structure exhibits a clear enhancement in the overall absorptivity along with a noticeably broader absorption bandwidth, demonstrating the ability of the TD3 model to improve both the magnitude and spectral extent of the device’s absorption performance. The optimized parameters and their corresponding absorption results compared to the reference design are summarized in Table 4. This confirms that the TD3-RL framework can efficiently optimize heterogeneous metasurface designs when guided by a consistent physical objective and reward structure. The results confirm the generalizability of the proposed method and its suitability for diverse classes of dielectric metamaterial absorbers.

**Table 4:** Design parameters and bandwidth comparison of the dielectric metasurface absorber between the TD3-RL results and that previously reported in ^52^.

Parameters (nm)	Ref ^52^	Proposed
r	145	147.5
h	110	125
t	30	24.5
a	400	407
Mean Absorptivity (%)	83.7%	92.6%
Bandwidth (μm)	0.39–2.09	0.6–2.9

**Fig. 5:**
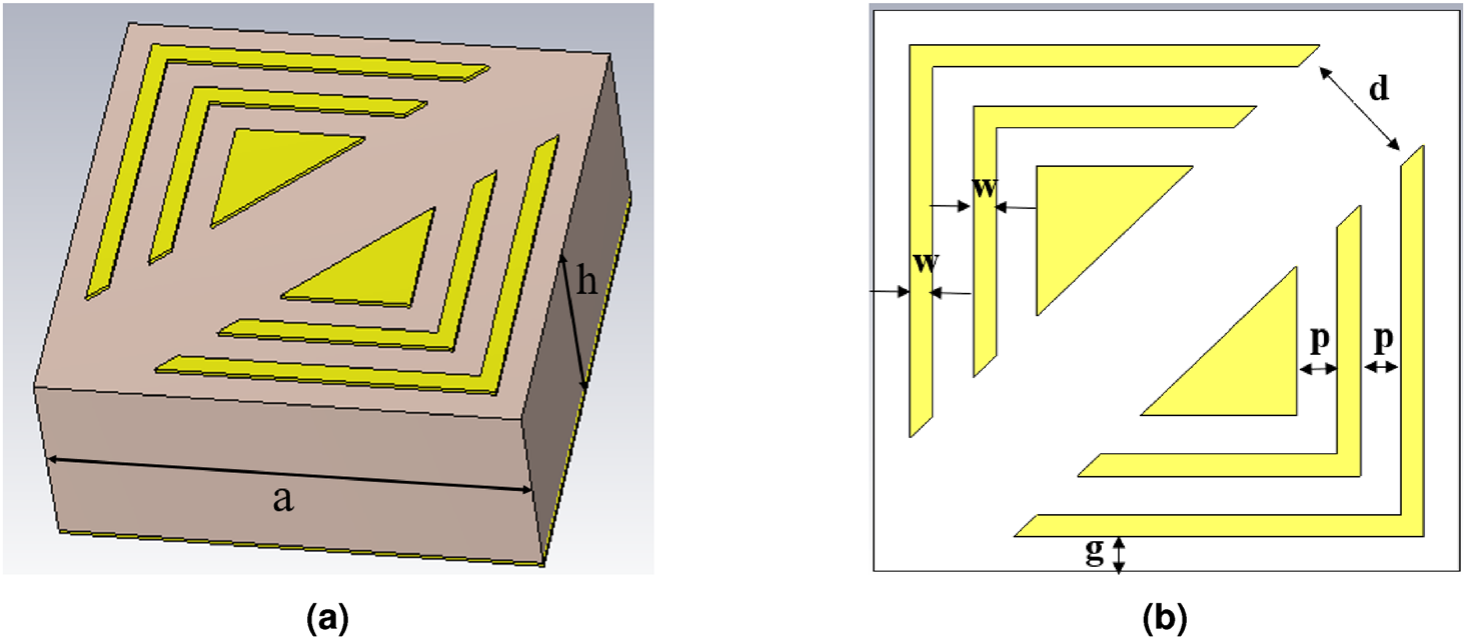
Design representations: (**a**) Three-dimensional structural overview and (**b**) Two-dimensional planar layout of the proposed metamaterial cross-polarizer.

Based on the successful results obtained from the previous metamaterial absorber optimization, the TD3-RL model is next employed to optimize a new broadband cross-polarization conversion metasurface (CPCM) structure. Figure  5 presents the structural layout of the proposed CPCM, where an additional triangular shape is added inside the L-shapes. The top resonator layer is composed of copper with constant conductivity $\sigma = 5.96 \times 10^{7}~\mathrm {S/m}$ and a thickness of 0.035 mm. The ground plane, which forms the bottom layer, is also made of copper with the same thickness, ensuring negligible transmission. A dielectric FR-4 substrate is placed between the top and bottom copper layers with a thickness of $h$ of 1.6 mm. The unit cell periodicity $a$ is set to 3.7 mm.

Similar to the previous study, d, g, w, and p are selected as design variables. The parameters are optimized within the following ranges: d $\in [0.1, 1]$, p $\in [0.1, 0.31]$, g $\in [0.1, 0.31]$, and w $\in [0.1, 0.31]$. The upper bound of d is reduced to prevent the introduced triangle from being removed during optimization. Periodic boundary conditions are used around the unit cell in the x- and y-directions. However, an open boundary condition is used along the propagation direction of the incident wave z-direction. Furthermore, the tetrahedral mesh of the structure is employed with an accuracy of $10^{-4}$ with a total number of elements of 143,938. The suggested CPCM is optimized using the TD3-RL model to obtain a wide-band cross polarizer.

In a reflective metamaterial cross-polarizer, the polarization conversion performance is commonly characterized using the co- and cross-polarized reflection coefficients. Under normal incidence, an *x*-polarized plane wave is excited toward the metasurface, and the reflected field can be decomposed into *x*- and *y*-polarized components. Accordingly, the co-polarized reflection coefficient is defined as $S_{xx}$, representing the reflected component maintaining the same polarization, whereas the cross-polarized reflection coefficient $S_{xy}$ represents the reflected component converted into the orthogonal polarization. Assuming negligible transmission due to the metallic ground plane, the polarization conversion ability is quantified by the polarization conversion ratio (PCR), defined as the fraction of the reflected power that is converted into the orthogonal polarization:

10 $$\begin{aligned} \textrm{PCR}=\frac{|S_{xy}|^2}{|S_{xx}|^2+|S_{xy}|^2}. \end{aligned}$$

A PCR approaching unity indicates near-perfect cross-polarization conversion, while lower values correspond to dominant co-polarized reflection.

**Fig. 6:**
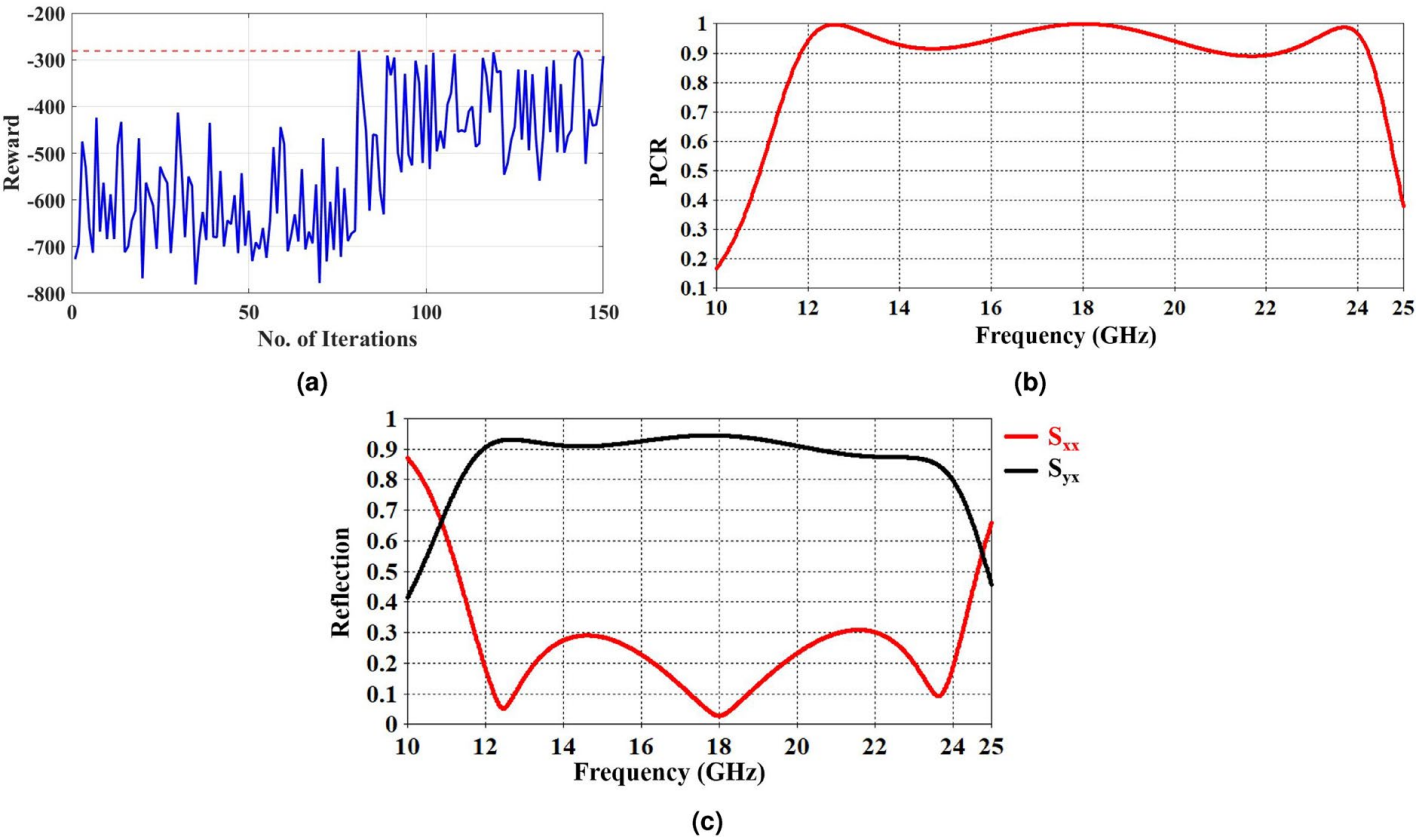
(**a**) The training chart for the TD3-RL model with Reward Vs No. of iterations (**b**) Polarization conversion ratio. (**c**) co- and cross-polarization components $S_{xx}$ and $S_{yx}$.

The same reward function in equation 6 is used but in this case y represents the fraction of the frequency spectrum with PCR exceeding 90% for 150 iterations. The number of iterations was raised to account for the greater structural complexity of the novel cross polarizer design, thereby providing the RL agent with sufficient opportunity to achieve improved optimization results. The initial parameters were chosen randomly, and the model got the best results after only 81 iterations. The whole training took 7 hours on the same workstation used in the first example. Figure  6a presents the reward values achieved by the proposed TD3-RL model across successive iterations, demonstrating the model’s ability to converge toward higher reward values. After applying the parameters obtained in the 81$^{\text {st}}$ iteration, Figures  6b and  6c present the PCR of the proposed unit cell and the corresponding reflection coefficients $S_{xx}$ and $S_{xy}$, respectively. The studied CPCM exhibits PCR greater than 90% over a broad frequency range from 11.8 to 24.2 GHz, including the full Ku-band and much of the K-band as shown in Fig. 6b. These results indicate that the proposed design holds strong potential for Ku-band applications. The device performance is summarized in Table 5.

**Table 5:** Design parameters and bandwidth comparison of the suggested CPCM.

Parameters (mm)	Initial	Proposed
d	0.4	0.925
g	0.19	0.23
w	0.25	0.145
p	0.12	0.255
Mean PCR (%)	68.1%	88%
Bandwidth (GHz)	13.8–17.3	11.8–24.2

**Fig. 7:**
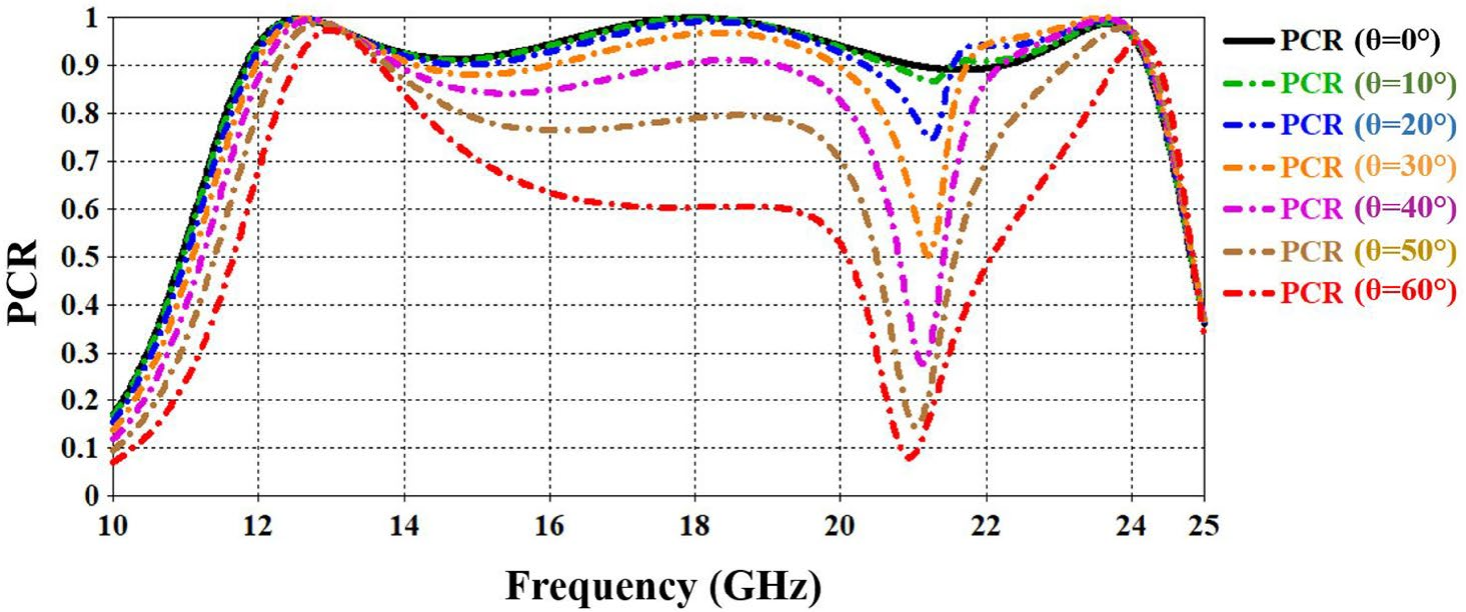
The impact of the incidence angle on the reflector response.

The stability and performance of the polarization-conversion metasurface can be evaluated by examining its effectiveness over a broad range of incidence angles. Figure  7 illustrates the response across different incident angles from 0°to 60°with PCR. The figure shows that till 20°incident angle the structure is fairly stable for the operating bandwidth with PCR more than 0.8. However, from 30°, 40°, and 50°the higher frequency band slightly decreases to 20.5 GHz, 20.1 GHz, and 19.3 GHz respectively while PCR more that 0.8. these results shows that the CPCM performs well under oblique incidence.

**Fig. 8:**
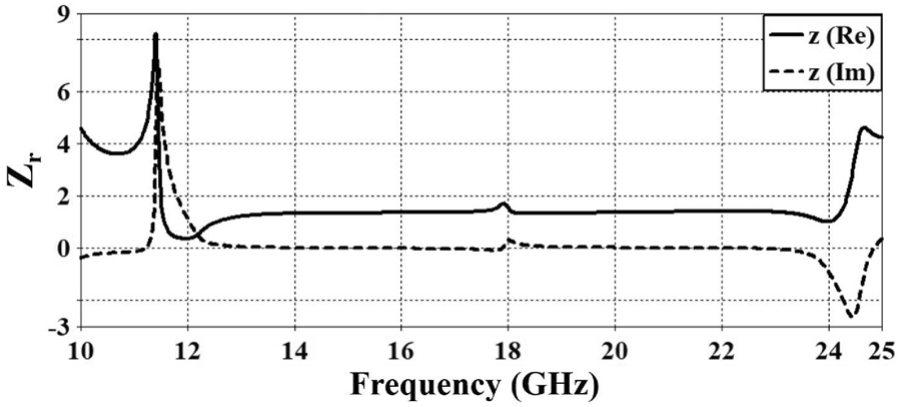
Relative impedance of the reported CPCM.

**Fig. 9:**
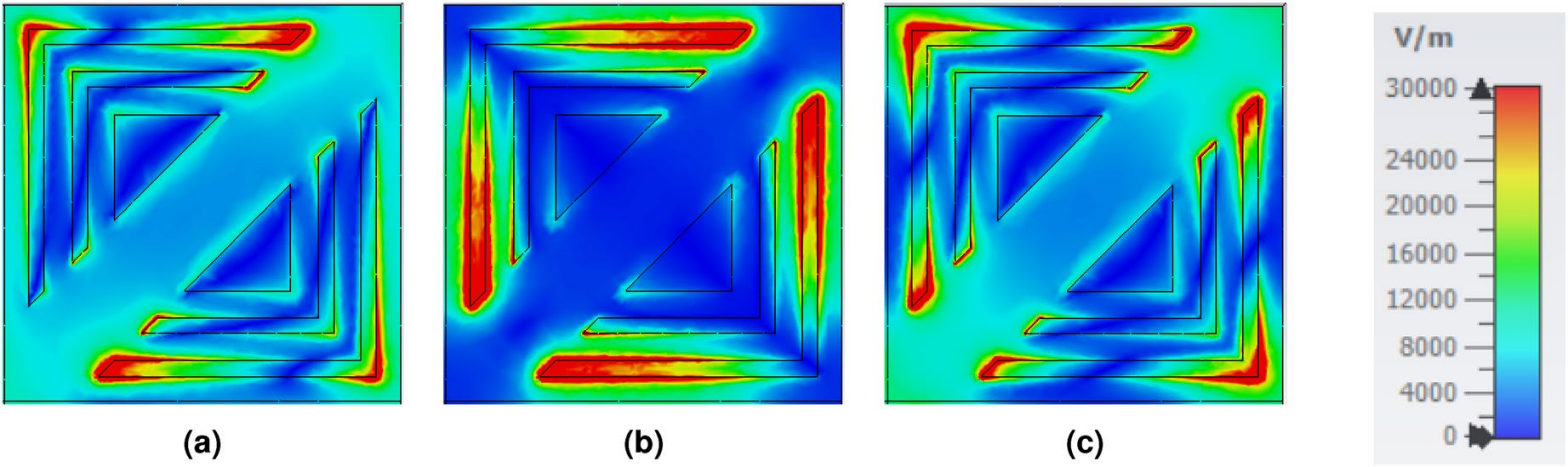
Simulated electric field distributions of the proposed MA at frequencies of (**a**) 12.5 GHz, (**b**) 18 GHz, and (**c**) 23.7 GHz.

**Fig. 10:**
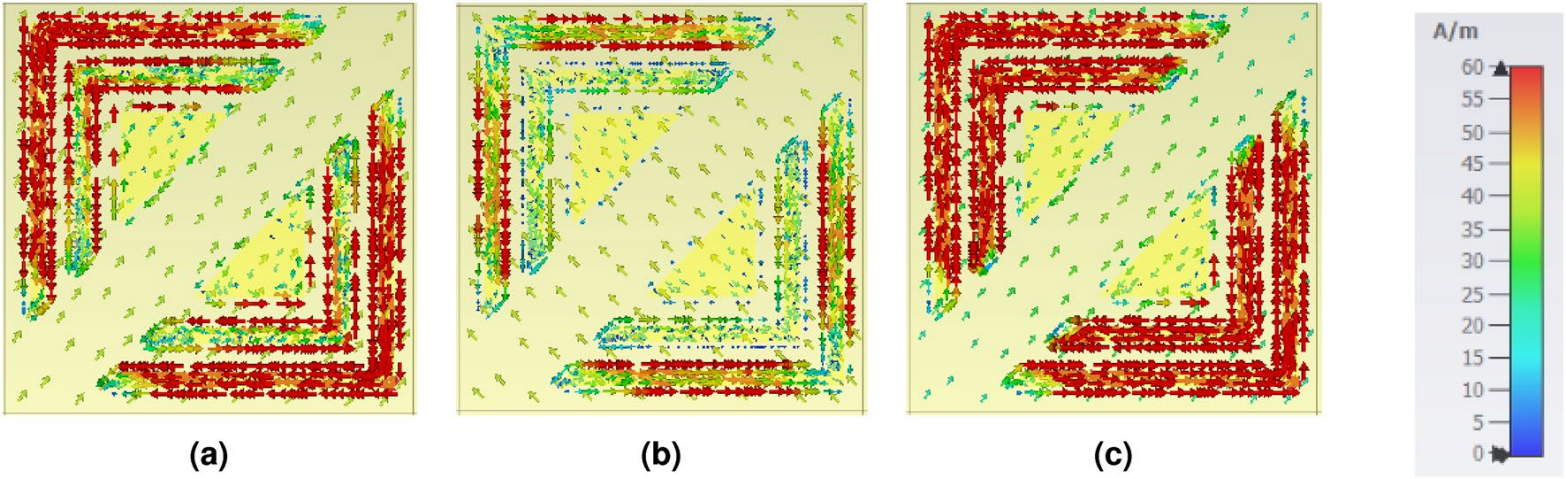
Simulated surface current density of the proposed design at frequencies of (**a**) 12.5 GHz, (**b**) 18 GHz, and (**c**) 23.7 GHz.

The physical origin of polarization conversion in the proposed metasurface can be understood through the impedance response and the corresponding electromagnetic field distributions at resonance. As shown in Fig. 8, the retrieved normalized input impedance exhibits a near-perfect matching condition within the operating band, where the real part approaches unity and imaginary part remains close to zero. This impedance matching significantly suppresses the co-polarized reflection component and enhances the excitation of resonant modes, which is essential for achieving efficient polarization conversion. To further elucidate the conversion mechanism, the electric field distributions at the three resonance frequency peaks–12.5 GHz, 18 GHz, and 23.7 GHz–are depicted in Fig. 9. Strong electric-field localization is observed along different arms and corners of the resonator, indicating the excitation of multiple anisotropic resonant modes and the formation of orthogonal field components. In addition, the corresponding surface current density distributions illustrated in Fig. 10 show pronounced non-collinear and rotational current paths along the metallic segments. These induced currents generate scattered fields with significant perpendicular polarization components, thereby producing strong cross-polarized reflected waves. Overall, the combined impedance matching behavior (Fig. 8) and the resonance-driven electric field/current distributions (Figs. 9-10) confirm that polarization conversion is primarily governed by anisotropic resonant coupling and current reorientation at the identified resonance frequencies.

To experimentally validate the performance of the proposed metamaterial cross-polarizer, a prototype is fabricated based on the optimized design parameters. It comprises $40 \times 40$ unit cells, forming a cuboid with dimensions of $148~\text {mm} \times 148~\text {mm} \times 1.6~\text {mm}$. Due to fabrication constraints, the resonator width w is adjusted to 0.2 mm. The final set of parameters used for fabrication is detailed in Table 6. The prototype is fabricated using standard photolithography techniques, and its performance is experimentally characterized using two linearly polarized standard-gain horn antennas connected to a vector network analyzer (Agilent FieldFox^®^ model N9918A) in a bi-static reflection configuration. The x- and y-polarized horns are connected to ports 1 and 2 of the VNA, respectively, enabling simultaneous measurement of co- and cross-polarized reflection components. The co-polarized reflectance is obtained from $S_{xx}$ and $S_{yy}$, while the cross-polarized reflection is extracted from $S_{yx}$. This measurement arrangement enables direct evaluation of polarization conversion by comparing the relative magnitudes of co- and cross-polarized reflected signals under identical experimental conditions.

**Table 6:** The value of the fabricated parameters of the proposed CPCM.

Parameter	h	a	g	p	w	d
Value (mm)	1.6	3.7	0.23	0.255	0.2	0.925

**Fig. 11:**
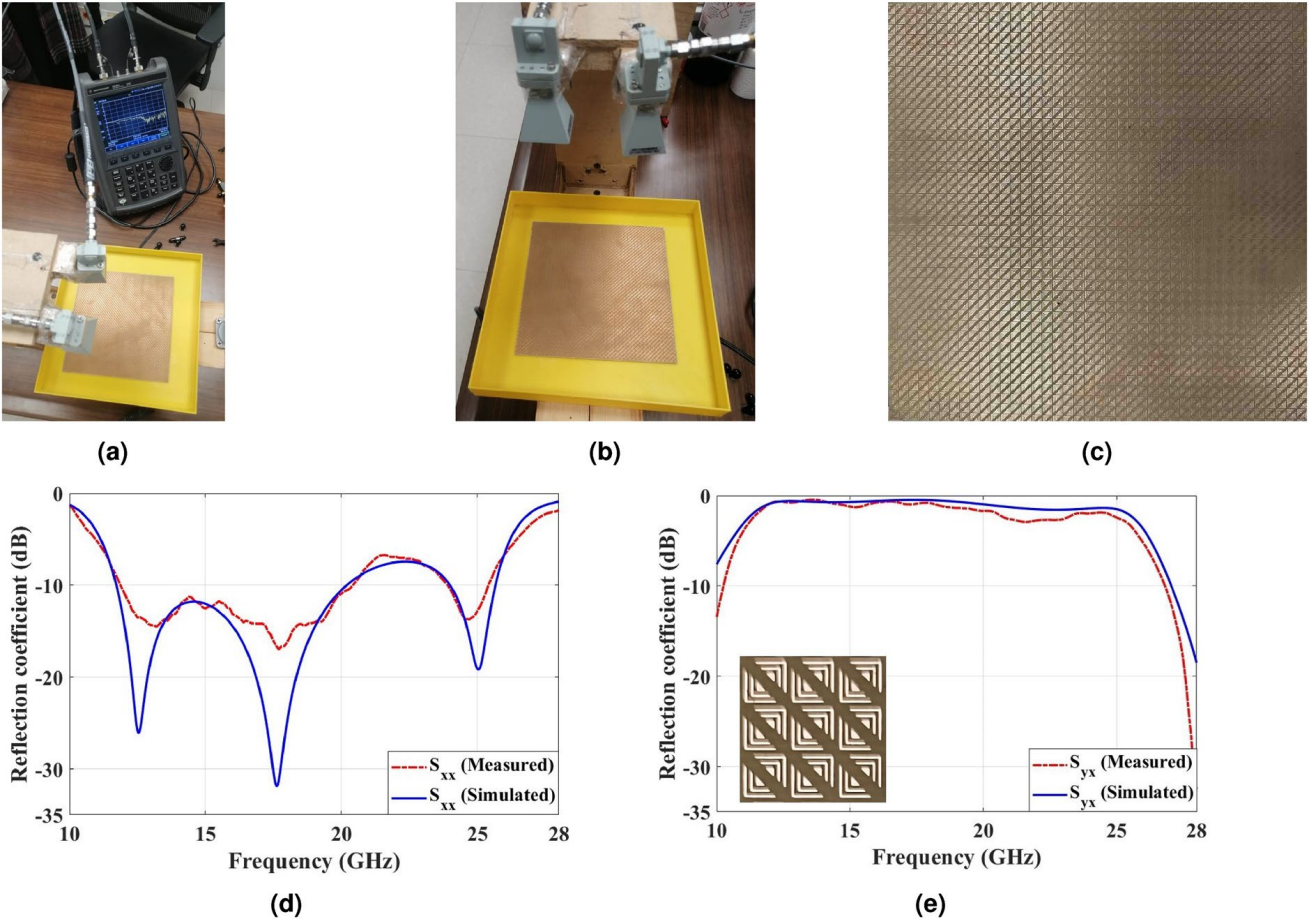
(**a**,**b**) Measurement setup, (**c**) Fabricated prototype, (**d**) simulation and measurement results of co-polarization reflection coefficient, and (**e**) simulation and measurement results of cross-polarization reflection coefficient.

The complete measurement setup is illustrated in Fig. 11a 11b, while the fabricated prototype is shown in Fig. 11c. A comparison between the simulated and measured reflectance coefficients are introduced in Fig. 11d for the co-polarization reflection and Fig. 11e for the cross-polarization reflection. As shown, the measured reflectance curve exhibits slight difference compared to its simulated counterpart. This discrepancy can be attributed to several factors. The finite size of the fabricated sample introduces edge diffraction and scattering effects that are absent in the idealized simulation, which assumes an infinitely periodic structure. Further, minor deviations in geometry or material properties may have occurred during the fabrication process, contributing to performance variation.

In Table 7, the performance of the proposed design is compared with various previously reported wide-band metamaterial cross-polarizers. This comparison shows that the reported design offers several advantages, including compact thickness, near-perfect cross-polarization reflection, and a wide bandwidth, all achieved without the use of multilayer structures or lumped elements.

**Table 7:** Performance comparison of the proposed design with reported designs in the literature.

Ref.	BW (GHz)	Unit Cell dimensions (mm)	Thickness (mm)	Bandwidth (PCR>0.9) (%)
Two L shapes with triangle resonators (This work)	**11.7–24.1**	**3.7** × **3.7**	**1.6**	**88**
Two concentric deformed rings resonator ^53^	8.75–17.75	6.3 $\times$ 6.3	2	68
Triple-arrow resonator ^54^	8–18.5	5 $\times$ 5	2.4	80
Double square ring ^55^	6.67–17.1	12 $\times$ 12	3.2	87.8
Angle-shaped resonator ^56^	8.6–22	8 $\times$ 8	3	88
Double-meander-line-shaped resonator ^57^	8.4–20.1	7 $\times$ 7	2.5	82.3
Chessboard resonator ^58^	7–16	8 $\times$ 8	3.04	78

## Conclusion

In conclusion, this study demonstrates the potential of reinforcement learning–specifically the TD3 algorithm–as a powerful and efficient tool for the inverse design of photonic structures. By enabling direct interaction with the simulation environment, the proposed RL-based framework effectively eliminates the need for gradient calculations or surrogate models. Its ability to rapidly converge to optimal solutions was validated through the optimization of an L-shaped resonator-based metamaterial absorber, achieving over 90% absorption across a wide frequency band in just 23 iterations. Furthermore, a novel CPCM design is successfully proposed, optimized, and fabricated, exhibiting high PCR over an even broader frequency range (11.8–24.2 GHz). The close agreement between simulation and measurement confirms the practical viability of the design and highlights the robustness of the TD3-RL approach. Overall, this work primarily advances the development of automated optimization frameworks based on reinforcement learning, laying the foundation for future research in this field.

## Data Availability

No datasets were used during this study as the TD3 reinforcement learning approach is an unsupervised optimization technique. All data generated or analyzed during this study are included in this published article. Additionally, correspondence and requests for simulation data can be addressed to M.F.O.H. and S.S.A.O.
